# Evaluation of a portable glucometer for blood glucose measurement in juvenile green sea turtles ( *Chelonia mydas* )

**DOI:** 10.1111/avj.70130

**Published:** 2026-08-12

**Authors:** YAA Pérez, MG Chenard, G Martinez‐Souza, VHS Ney, K Russano, J de Menezes Paes, VZ Lemos, BA de Freitas, KM de Souza, JVM da Silva, T Gião, LV dos Santos, C de Freitas Campos, MA Helayel, ET Mársico, NX de Alencar, KVGC da Silva

**Affiliations:** ^1^ Faculdade de Veterinária Universidade Federal Fluminense Niterói Brazil; ^2^ Instituto Caminho Marinho Itapirubá Brazil; ^3^ Laboratório de Biometria e Conservação, Instituto de Matemática, Estatística e Física Universidade Federal do Rio Grande Rio Grande Brazil; ^4^ Instituto de Matemática e Estatística Universidade de São Paulo São Paulo Brazil; ^5^ Programa de Pós‐Graduação em Oceanografia Universidade Federal de Santa Catarina Florianópolis Brazil; ^6^ Programa de Pós‐graduação em Ambientometria, Laboratório de Biometria e Conservação, Instituto de Matemática, Estatística e Física Universidade Federal do Rio Grande Rio Grande Brazil; ^7^ Instituto de Ciência e Tecnologia em Biomodelos Fundação Oswaldo Cruz Rio de Janeiro Brazil

**Keywords:** fibropapillomatosis, glycaemia, marine turtle, methods comparison, point‐of‐care device, Southeastern Brazil

## Abstract

Blood glucose is a key physiological indicator in sea turtles, but its measurement in field settings can be challenging. Portable point‐of‐care (POC) glucometers offer practicality, yet their agreement with reference laboratory methods has not been established for juvenile green sea turtles (*Chelonia mydas*) from the Southwestern Atlantic. We compared glucose concentrations from a portable glucometer with a reference enzymatic method using blood samples from 30 free‐ranging juvenile green turtles captured in Rio de Janeiro, Brazil. Agreement was assessed by Wilcoxon signed‐rank test, Passing‐Bablok regression and Bland–Altman analysis. The influence of fibropapillomatosis and packed cell volume was also examined. The glucometer significantly underestimated glucose compared to the reference method (median 70 vs. 90 mg/dL, respectively, P < 0.001), with a mean bias of −17.9 mg/dL and wide limits of agreement. Passing‐Bablok regression showed no significant systematic or proportional errors (95% CI for intercept included 0; 95% CI for slope included 1). Only 43% of glucometer readings fell within ±20% of the reference ranges reported for sea turtles. Packed cell volume correction slightly reduced the bias but did not improve agreement. Fibropapillomatosis status did not affect absolute glucose concentrations but influenced relative bias when packed cell volume was within the manufacturer's validated range. The portable glucometer is not interchangeable with the reference method for this species. While it may provide rapid field estimates, its results require cautious interpretation and, whenever possible, laboratory validation.

## Introduction

Sea turtles are long‐lived species with a global distribution. Some species, such as green sea turtles (*Chelonia mydas*), occupy coastal regions with dense human populations during their juvenile stages, raising concerns about their health and conservation.^1^ Proactive monitoring of aggregation areas, feeding grounds and developmental habitats using diverse biomarkers can help identify potential threats.^2^, ^3^

Blood biochemistry monitoring in free‐ranging sea turtles plays an important role in understanding their physiology and health, supporting the use of biomarkers to monitor populations under different environmental conditions.^2^, ^3^ Among these parameters, blood glucose is a key indicator of metabolic and physiological status in vertebrates. In reptiles, including sea turtles, glucose dynamics are not yet fully understood and may be influenced by multiple factors.^4^, ^5^ Accurate assessment of blood glucose is therefore essential for evaluating the health of sea turtle populations, as it provides insight into metabolic processes under various conditions, including stress responses, environmental influences and disease states.^6^, ^7^, ^8^

Portable point‐of‐care (POC) devices are widely used in field studies due to their practicality, facilitating logistics, reducing costs and providing rapid results.^9^, ^10^ Despite the availability of established laboratory methods for blood glucose determination, including dry chemistry analysis, bichromatic spectrophotometry and reflectance photometry, interest in portable devices has increased.^9^, ^10^, ^11^, ^12^

Recent studies have investigated the agreement between POC devices and traditional laboratory methods across various species, including dogs and cats^13^, ^14^; small ruminants and cattle^15^, ^16^, ^17^; and wildlife species such as rhinoceros auklets (*Cerorhinca monocerata*),^18^ white‐tailed deer (*Odocoileus virginianus*)^19^ and reptiles including bearded dragons (*Pogona vitticeps*), snakes and tortoises.^20^ However, this agreement can vary depending on the device, methodology and species.^5^, ^9^, ^11^ Some studies have evaluated the use of POC devices to measure glucose in green turtles (*Chelonia mydas*) Perrault et al.,^11^ loggerhead (*Caretta caretta*) Kunze et al.^5^ and Kemp's ridley (*Lepidochelys kempii*) Perrault et al.,^9^ providing valuable information for researchers and conservationists. However, in reptiles, POC devices do not always yield satisfactory results when compared to laboratory‐based clinical chemistry analysers.^20^, ^21^

In the Southwestern Atlantic Ocean, juvenile green turtles frequently inhabit coastal areas subject to high anthropogenic pressure. Individuals in these environments, such as Guanabara Bay in Rio de Janeiro, may exhibit changes in physiological parameters, including blood glucose concentrations, reflecting environmental influences on metabolic status. In addition, these populations are commonly affected by fibropapillomatosis, although the potential influence of this disease on glucose dynamics remains poorly understood. In Brazil, research on blood glucose concentrations in juvenile green turtles, the potential influence of fibropapillomatosis on glucose dynamics, and the performance of POC devices is limited. Such investigations are particularly relevant because geographic and seasonal variations, as well as the type of POC device used, may influence analyte concentrations and contribute to differences in agreement between methods.

Therefore, differences between POC and laboratory‐based glucose measurements may exist in juvenile green turtles and that fibropapillomatosis could contribute to variability in glucose concentrations. Accordingly, the aim of this study was to evaluate the agreement between a portable point‐of‐care glucometer and a reference laboratory method for blood glucose determination in juvenile green turtles from the Guanabara Bay in Rio de Janeiro, Brazil, and to explore the potential influence of fibropapillomatosis on glucose concentrations.

## Materials and methods

### Study areas and captures

Thirty free‐ranging green turtles were captured in three areas of Rio de Janeiro, Brazil: Marina da Glória (−22.917, −43.173), Fortaleza de São João (−22.943, −43.158) and Clube Naval de Charitas (−22.934, −43.107), during a 1‐week expedition, with sampling conducted at 24‐h intervals. Capture procedures consisted of shore‐deployed nets, set passively or as partial encirclement. Trained research divers monitored nets continuously, disentangled turtles quickly and transferred them to the shore team within 1–2 min.

All turtles were handled according to ethical guidelines and released after sampling and data collection. Procedures were approved by the Animal Use Ethics Committee (CEUA) of the Universidade Federal Fluminense (protocol no. 8308100221) and by the Biodiversity Authorization and Information System (SISBIO, no. 78601). Laboratory procedures were conducted at the Faculdade de Veterinária, Universidade Federal Fluminense, Niterói, Rio de Janeiro.

### Data collection and sampling

A standardised sampling order was followed to preserve sample quality. First, turtles were photo‐identified and measured: Mass and standard curved carapace length (CCL) were recorded. The body condition index (BCI) was calculated according to Bjorndal et al.^22^ Life‐stage class was classified based on CCL following Chaloupka and Limpus.^23^

After a rest period of approximately 10–15 min to reduce acute capture stress effects, blood was collected from the external jugular vein using disposable syringes (10 mL) and needles (21G). The sampled volume did not exceed 3 mL/kg.^24^ Following blood sampling, the presence of fibropapilloma tumors was recorded based on tumor size and number.^25^

### Glucose measurements and portable glucometer

After blood sampling, an aliquot of whole blood was used to measure glucose with a glucometer (Accu‐Chek Active®, Roche Diabetes Care). Measurements were performed within the manufacturer's recommended operating temperature range (8°C–42°C), under field conditions ranging from approximately 9°C to 31.5°C. Test strips were stored and handled according to the manufacturer's guidelines and allowed to reach ambient temperature before use. From the remaining blood volume, 5 mL were stored in fluoride‐containing tubes at 4°C and transported to the laboratory for plasma glucose determination. Glucose concentrations in plasma were determined using commercial enzymatic colorimetric assay (Labtest®) on a previously calibrated Labmax 240 Premium automatic spectrophotometer.^26^, ^27^ The remaining sample volume was used for packed cell volume and subsequent analyses not included in this manuscript.

The Accu‐Chek Active® system uses a glucose dehydrogenase‐based enzymatic method and provides plasma‐equivalent glucose concentration despite using whole blood. The analytical measurement range is 10–600 mg/dL, with a validated human packed cell volume range of 20–55%. The device is calibrated against the hexokinase reference method and complies with ISO 15197:2013 accuracy standards.

### Statistical analysis

All statistical analyses were performed in R (version 4.5). Data distribution was assessed using the Shapiro–Wilk test. As paired glucose measurements did not follow a normal distribution, nonparametric methods were used where appropriate. Difference between the glucometer and the reference enzymatic method was evaluated using the Wilcoxon signed‐rank test for paired comparisons. Absolute bias (absolute bias = glucometer − enzymatic method) and relative bias (relative bias = [(glucometer − enzymatic method) / enzymatic method] × 100) were calculated for each sample.

Although the portable glucometer provides plasma‐equivalent glucose concentration, packed cell volume correction was applied to evaluate potential residual bias. Corrected values were calculated using the plasma‐equivalent adjustment recommended by the American Society for Veterinary Clinical Pathology (ASVCP)^28^: glucometer corrected = glucometer / [1 − (0.0024 × packed cell volume)]. This correction was used for exploratory purposes and was not considered the primary analytical outcome.

Agreement between methods was assessed using Passing‐Bablok regression and Bland–Altman bias, including calculation of mean bias and limits of agreement (LOA = mean bias ±1.96 SD). Clinical acceptability was explored by calculating the percentage of measurements within ±20% of the reference method (i.e. enzymatic method), and observed total analytical error (TEobs) was estimated. To assess the potential influence of fibropapillomatosis on method agreement, relative bias was compared between turtles affected by fibropapillomatosis and turtles not affected by fibropapillomatosis using the Wilcoxon rank‐sum test. Additionally, due to potential biological differences in glucose concentrations, enzymatic method and glucometer concentration were also compared between turtles with and without fibropapillomatosis using the Wilcoxon rank‐sum test.

A sensitivity analysis was performed by restricting the dataset to samples within the manufacturer's validated packed cell volume range (20–55%) to evaluate the robustness of results under optimal analytical conditions. Statistical significance was set at P < 0.05.

## Results

All captured green turtles were classified as juveniles (< 65 cm CCL), and sex was not determined. The median glucose measured by the glucometer was 70 mg/dL (range: 42–118 mg/dL), while the median reference enzymatic method was 90 mg/dL (range: 62–122 mg/dL). Packed cell volume‐corrected glucometer concentrations had a median of 75 mg/dL (range: 49–130 mg/dL). Body mass ranged from 2.7 to 18.1 kg, carapace length (CCL) from 28.3 to 52.6 cm and BCI from 0.99 to 1.32. Packed cell volume ranged from 10 to 38% (Table 1). Fibropapillomatosis was present in 40% (n = 12) of the turtles. Among affected turtles, 75% (n = 9) were classified as mild, 8.38% (n = 1) as moderate and 16.7% (n = 2) as severe.

**Table 1:** Descriptive analysis of biometrics, glucose concentrations and packed cell volume values of green sea turtles (*Chelonia mydas*) with and without fibropapillomatosis in Rio de Janeiro, Brazil

	Non‐FP turtles					FP turtles				
	N	Mean ± SD	Median	Min – Max	Reference	N	Mean ± SD	Median	Min – Max	Reference
Mass (kg)	18	6.2 ± 4.1	5.2	3–18.1	‐	12	8.1 ± 3.2	8.2	2.7–15.1	‐
CCL (cm)	18	36.6 ± 6.3	36.1	28.3–52.6	‐	12	41.0 ± 5.9	42.0	28.8–51.7	‐
BCI	18	1.15 ± 0.08	1.15	0.99–1.32	‐	12	1.12 ± 0.06	1.1	1.05–1.26	‐
PCV (%)	18	29 ± 7	30	10–38	28 ± 8^29^	10	26 ± 6.0	27	13–36	24 ± 8^29^
Glucose glucometer (mg/dL)	18	73 ± 18	69	45–118		12	69 ± 16	70	42–96	
Glucose enzymatic method (mg/dL)	18	87 ± 16	88	62–122	113 [64–207]^30^ 107 (7)^31^ 72 (11)^32^	12	91 ± 19	91	66–116	90–131^30^ 118 (6)^31^ 72 (8)^32^

Mean ± standard deviation,^29^ Mean [min. − max.],^30^ Mean (standard error),^31^ and Mean ± standard deviation.^32^

BCI, body condition index; CCL, curved carapace length; FP, fibropapillomatosis; Max, maximum; Min, minimum; PCV, packed cell volume; SD, standard deviation.

The distribution of absolute bias was not normal (Shapiro–Wilk *W* = 0.903, P = 0.010), and nonparametric analyses were therefore applied. The Wilcoxon signed‐rank test revealed a significant difference between the glucometer and the enzymatic method measurements (*V* = 17, P < 0.001), confirming a systematic negative bias of the glucometer relative to the reference method.

The glucometer readings showed a strong correlation with the reference method (Spearman's *r*_s_ = 0.85, P < 0.001). However, Passing‐Bablok regression did not show statistically significant systematic and proportional errors, as the 95% confidence intervals included 0 and 1, respectively (Intercept 95% CI: −32.42 to 7.09; Slope 95% CI: 0.68 to 1.17). Bland–Altman bias was −17.9 mg/dL, with wide limits of agreement (mean ± 1.96 SD), highlighting notable variability between methods (Figure 1). Median relative bias did not differ significantly between turtles affected and not affected by fibropapillomatosis (Wilcoxon rank‐sum test, *W* = 152, P = 0.066), indicating that fibropapillomatosis status did not significantly influence the agreement between methods. Clinical acceptability was limited, with only 43.3% of measurements within ±20% of enzymatic method concentrations.

**Figure 1:**
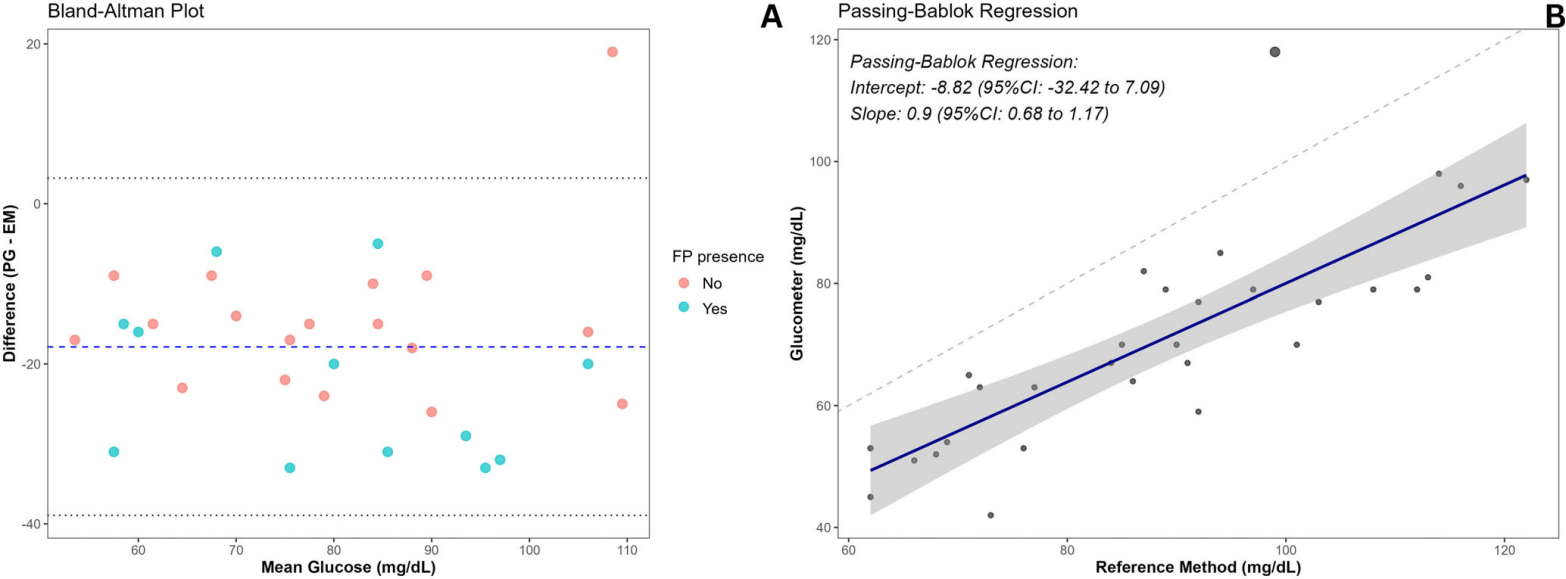
(A) Bland–Altman bias comparing paired glucose concentrations from the portable glucometer (PG) and the reference enzymatic method (EM). The blue dashed line represents the mean bias (−17.9 mg/dL), and the black dashed lines represent the 95% limits of agreement. (B) Passing‐Bablok regression comparing PG and EM measurements. The solid line is the regression line, and the dashed line represents the line of identity.

The glucometer corrected values showed a reduced mean bias (−12.7 mg/dL) compared to uncorrected glucometer (−17.9 mg/dL), although substantial disagreement with the reference method persisted. The Wilcoxon signed‐rank test for glucometer corrected showed a significant difference with enzymatic method measurements (*V* = 28, P < 0.001).

When the analysis was restricted to samples within the manufacturer's validated packed cell volume range (20–55%), the mean bias remained similar (−18.2 mg/dL) and agreement between methods did not improve. In this case, the Wilcoxon signed‐rank test was still significant (*V* = 15, P < 0.001). However, under these conditions, relative bias differed significantly between turtles with and without fibropapillomatosis (Wilcoxon rank‐sum test, *W* = 127, P = 0.007), suggesting that fibropapillomatosis status may influence glucometer performance when packed cell volume‐related variability is minimised.

No significant differences in glucose concentrations were observed between turtles with and without fibropapillomatosis for either the reference enzymatic method (*W* = 94.5, P = 0.582) or the glucometer (*W* = 115, P = 0.783). Clinical acceptability remained low, with only 42.3% of measurements within ±20% of EM values.

## Discussion

In this study, glucose concentrations determined using a point‐of‐care device (Accu‐Chek Active®) were compared with an automated spectrophotometric laboratory method. The paired Wilcoxon signed‐rank test demonstrated a significant difference between methods, confirming a systematic negative bias of the glucometer relative to the reference enzymatic method. Moreover, the glucometer tended to produce lower glucose concentrations (~18 mg/dL) than the enzymatic method, and this difference was variable across samples.

The green turtles included in this study were juveniles,^23^ with a BCI within the normal to robust range.^33^ Packed cell volume parameters were within or close to the ranges previously reported for green turtles from southeastern Brazil. Fibropapillomatosis was observed in 40% of the turtles, a relatively high frequency compared to previous reports in Brazil.^29^, ^34^, ^35^ However, given the limited sample size, this prevalence should be interpreted with caution and cannot be extrapolated to the broader population.

Measured blood glucose concentrations were comparable to those reported previously in Brazil,^30^ although turtles affected by fibropapillomatosis in the present study tended to show values closer to the lower range described by those authors. Overall, blood glucose concentrations in both turtles with and without fibropapillomatosis were consistent with previous reports in green turtles, although individual values overlapped the ranges described for turtles with and without fibropapillomatosis.^29^, ^30^, ^31^, ^32^

Similar underestimation of blood glucose concentrations by glucometers has been reported in other chelonian species. In previous studies involving *Emys marmorata* and *Trachemys* spp., the Accu‐Chek device has been used for glucose determination.^36^ However, validation studies in *Trachemys* spp. reported low accuracy and a systematic underestimation of glucose concentration.^21^ This pattern is consistent with previous evidence indicating a mean underestimation of approximately 20 mg/dL in reptiles when using portable glucometers.^21^, ^37^

Packed cell volume correction^28^ reduced the mean bias but did not eliminate disagreement, and restricting the analysis to the manufacturer's validated packed cell volume range (20–55%) also failed to improve agreement, indicating that packed cell volume alone does not fully explain the observed bias. Although packed cell volume can influence glucose measurements, particularly at extreme values,^5^, ^9^ species‐specific factors likely contribute to the observed discrepancies. In cattle, high packed cell volume has been associated with falsely low glucose readings using Accu‐Chek,^16^ whereas this effect was not observed in chelonians,^21^ suggesting important physiological differences.

Furthermore, portable glucometers are calibrated using mammalian blood characteristics, including algorithms that convert whole‐blood glucose to plasma‐equivalent values, which may not accurately reflect reptilian physiology because of nucleated erythrocytes and differences in glucose metabolism.^5^, ^9^, ^28^ Previous studies in sea turtles have therefore emphasised the need for species‐ and device‐specific validation and the establishment of method‐specific reference intervals. Consistent with this recommendation, Perrault and others^9^ established reference intervals for the TRUEtrack glucometer in immature Kemp's ridleys and emphasised interpretation using device‐specific reference intervals together with concurrent packed cell volume data.^5^, ^9^

Although no significant differences in glucose concentrations were observed between turtles with and without fibropapillomatosis using either method, this finding is consistent with previous reports that found no relationship between fibropapillomatosis status and glucose concentrations.^30^, ^32^ However, other authors have reported variable patterns depending on environmental or stress‐related conditions.^6^, ^31^ Interestingly, when restricted to samples within the validated packed cell volume range, relative bias differed significantly between turtles with and without fibropapillomatosis, despite the absence of differences in absolute glucose concentrations. This suggests that disease‐related physiological alterations may influence glucometer performance independently of packed cell volume, rather than directly affecting systemic glucose concentration. To our knowledge, this is the first study to report such an interaction.

Clinical acceptability remained low in all analyses, with less than half of the results meeting the ±20% acceptability criterion proposed by Gerber and Freeman.^28^ Despite the device's compliance with ISO 15197:2013 standards in human samples, its performance in sea turtles was substantially less accurately, likely due to physiological differences and preanalytical factors such as capture stress and sample handling.^5^, ^9^, ^12^ Although packed cell volume correction reduced the mean bias, substantial disagreement persisted, supporting the conclusion that the portable glucometer cannot be considered clinically interchangeable with the reference enzymatic method.

Overall, these findings indicate that the portable glucometer cannot be considered a clinically interchangeable substitute for the reference method in juvenile green turtles. While it may still provide rapid, approximate estimates under field conditions and could be useful for identifying markedly elevated glucose concentrations associated with acute physiological stress, its results should be interpreted with caution and, whenever possible, complemented by laboratory‐based analyses.

In conclusion, the portable glucometer showed limited agreement with the reference enzymatic method and low clinical acceptability in juvenile green turtles from Guanabara Bay. Although it may provide rapid estimates under field conditions, its use should be approached with caution, particularly in individuals with fibropapillomatosis. Therefore, it cannot be considered a direct substitute for laboratory‐based methods and should be used only as a complementary tool.

## Conflicts of interest and sources of funding

This study was financed in part by the Coordenação de Aperfeiçoamento de Pessoal de Nível Superior‐Brasil (CAPES) and Fundação Owaldo Cruz (FIOCRUZ).

## Data Availability

The data that support the findings of this study are available on request from the corresponding author. The data are not publicly available due to privacy or ethical restrictions.
